# Insights into Elemental Composition and Sources of Fine and Coarse Particulate Matter in Dense Traffic Areas in Toronto and Vancouver, Canada

**DOI:** 10.3390/toxics9100264

**Published:** 2021-10-14

**Authors:** Valbona Celo, Mahmoud M. Yassine, Ewa Dabek-Zlotorzynska

**Affiliations:** Analysis and Air Quality Section, Air Quality Research Division, Science and Technology Branch, Environment and Climate Change Canada, 335 River Road, Ottawa, ON K1A 0H3, Canada; valbona.celo@canada.ca (V.C.); mahmoud.yassine@canada.ca (M.M.Y.)

**Keywords:** PM_2.5_, PM_10-2.5_, non-exhaust emissions, trace metals, source apportionment, PMF

## Abstract

Traffic is a significant pollution source in cities and has caused various health and environmental concerns worldwide. Therefore, an improved understanding of traffic impacts on particle concentrations and their components could help mitigate air pollution. In this study, the characteristics and sources of trace elements in PM_2.5_ (fine), and PM_10-2.5_ (coarse), were investigated in dense traffic areas in Toronto and Vancouver, Canada, from 2015–2017. At nearby urban background sites, 24-h integrated PM samples were also concurrently collected. The PM_2.5_ and PM_10-2.5_ masses, and a number of elements (i.e., Fe, Ba, Cu, Sb, Zn, Cr), showed clear increases at each near-road site, related to the traffic emissions resulting from resuspension and/or abrasion sources. The trace elements showed a clear partitioning trend between PM_2.5_ and PM_10-2.5_, thus reflecting the origin of some of these elements. The application of positive matrix factorization (PMF) to the combined fine and coarse metal data (86 total), with 24 observations at each site, was used to determine the contribution of different sources to the total metal concentrations in fine and coarse PM. Four major sources were identified by the PMF model, including two traffic non-exhaust (crustal/road dust, brake/tire wear) sources, along with regional and local industrial sources. Source apportionment indicated that the resuspended crustal/road dust factor was the dominant contributor to the total coarse-bound trace element (i.e., Fe, Ti, Ba, Cu, Zn, Sb, Cr) concentrations produced by vehicular exhaust and non-exhaust traffic-related processes that have been deposited onto the surface. The second non-exhaust factor related to brake/tire wear abrasion accounted for a considerable portion of the fine and coarse elemental (i.e., Ba, Fe, Cu, Zn, Sb) mass at both near-road sites. Regional and local industry contributed mostly to the fine elemental (i.e., S, As, Se, Cd, Pb) concentrations. Overall, the results show that non-exhaust traffic-related processes were major contributors to the various redox-active metal species (i.e., Fe, Cu) in both PM fractions. In addition, a substantial proportion of these metals in PM_2.5_ was water-soluble, which is an important contributor to the formation of reactive oxygen species and, thus, may lead to oxidative damage to cells in the human body. It appears that controlling traffic non-exhaust-related metals emissions, in the absence of significant point sources in the area, could have a pronounced effect on the redox activity of PM, with broad implications for the protection of public health.

## 1. Introduction

Air pollution continues to be one of the most pressing problems in urban areas because of the increased risk for a number of adverse health effects [1,2]. Particularly, exposure to ambient particulate matter (PM) emitted from traffic heavy roadways has been known to impact human health, from respiratory and cardiovascular diseases through to neurodegenerative effects [3,4,5,6]. Hence, characterizing the properties of traffic-related PM is crucial to understand the link between PM exposure and the health effects among populations living, working, or commuting in traffic-congested areas [1,4].

Traffic-generated particulate matter arises from vehicular exhaust and non-exhaust sources [7,8,9,10,11]. The former is related to fuel combustion, and the latter are due to the wear of brake systems (pads and discs), tires, road surface abrasion, and the resuspension of road dust particles. The latter matrix is composed of natural and anthropogenic materials that accumulate on the road surface and can be resuspended by wind and traffic flow. While numerous regulations and technological upgrades in the automotive field have succeeded in significantly reducing vehicle exhaust PM emissions in recent years, non-exhaust PM emissions are unaffected by such actions. Although there is consensus that vehicular exhaust emissions produce particles mainly in the fine fraction [8,9,10,11], non-exhaust emissions contribute to both fine and coarse modes of PM_10_, with the coarse fraction (particles with an aerodynamic diameter of 2.5–10 μm) being predominant [9,10,12]. Thus, the proportion of PM emissions from vehicular non-exhaust sources has rapidly increased in recent years, and become more and more relevant to health [9,10]. A portion of these health impacts is associated with inhalable PM and its metal content. Despite their low abundance compared to other species, evidence suggests metals have an enhanced toxicity compared to other compounds [13,14,15]. A number of recent studies have shown that ambient metal elements (in their soluble and insoluble forms) have been extensively associated with the generation of reactive oxygen species (ROS), which has been postulated to be an important mechanism leading to PM-induced toxicity and the associated adverse health effects [16,17,18,19,20,21]. In particular, sources with high concentrations of transition metals, such as Fe, Cu, Ba, Zn, Mn, Cr, and Ti, arising from non-exhaust emissions, appear to have a higher intrinsic oxidative potential (i.e., OP per microgram of PM) than other sources of PM [21,22,23,24,25,26,27,28,29]. In a recent population-based cohort study in Toronto, Canada, Zhang et al. [21] found that long-term exposure to Fe and Cu in PM_2.5_, and their combined impact on ROS, were consistently associated with increased cardiovascular disease deaths. Therefore, the study of the sources, transport, and spatiotemporal characteristics of these redox-active metals becomes of paramount importance, as this information will help guide the development of future policies for more effective pollutant control strategies. As a result, there have been more recent efforts to characterize traffic-related pollution, including non-exhaust emissions, which is a major concern in cities around the world [26,30,31,32,33,34,35,36,37,38,39,40,41,42,43].

The principal aim of the present study is to assess the levels and sources of atmospheric trace elements in the vicinity of a busy highway in Toronto, and beside a major trucking route in Vancouver. Part of the data presented in this study is included in our previous publication, where the chemical composition and sources of PM_2.5_ at the same roadways and nearby urban background areas in Toronto and Vancouver are reported [42]. This paper, therefore, focuses on aspects not previously covered by performing a comprehensive characterization of a wide suite of elements in PM_2.5_ (FPM = fine PM) and PM_10-2.5_ (CPM = coarse PM) collected during 2015–2017. The assessment of the levels of trace metals in coarse particles in near-road environments is important in understanding the potential health implications to a given population (i.e., potentially dangerous chronic exposure of pedestrians), since coarse particles often originate from local sources and are known to be geographically heterogeneous, in contrast to fine particles, which are more homogeneously dispersed over space. For example, there is some evidence that coarse particles may lead to oxidative stress and inflammation, as well as increased levels of cardiovascular and cerebrovascular mortality [23,44,45,46]. The water solubility of PM_2.5_-bound metals is also reported. The possible sources of metals, and their contributions to total FPM- and CPM-bond metal concentrations, at both near-road urban sites, were identified using the positive matrix factorization (PMF) model.

## 2. Experimental

### 2.1. Sampling Sites

**Figure 1:**
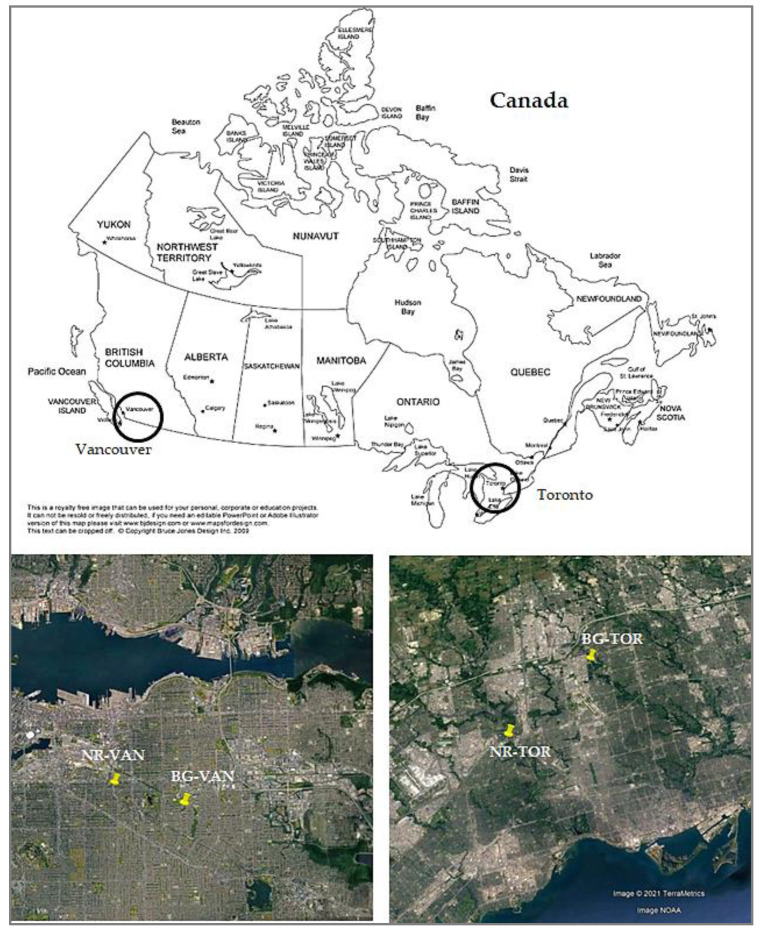
Map showing the sampling locations.

The measurements took place in two Canadian metropolises: Toronto and Vancouver (Figure 1). In Toronto, the near-road site was located south of the eastbound lanes of Highway 401, the busiest highway in North America (hereafter referred to as NR-TOR). In Vancouver, samples were collected at a traffic-heavy site, which is a known major truck route and one of the primary routes for goods travel from the south of Vancouver to the Port in the north (hereafter referred to as NR-VAN). Basic site information and the sampling periods are summarized in Table 1. Detailed descriptions of the NR-TOR and NR-VAN sites is provided in previous publications [40,42,47]. Samples were also collected at nearby background sites in Toronto (BG-TOR) and in Vancouver (BG-VAN), located away from roads. For the near-road sites, the wind frequently came from the direction of the roadway, with higher wind speeds observed at NR-VAN. The mean (range) ambient temperature and relative humidity during this period were +10 ± 11 °C (−25 to +35 °C) and 63 ± 16% for Toronto, respectively, and +12 ± 7 °C (−8 to +33 °C) and 72 ± 16% for Vancouver, respectively [47].

### 2.2. Sampling and Elemental Analysis

Integrated PM samples were collected on 47-mm id polytetrafluoroethylene (PTFE) filters (Pall Corporation, New York, NY, USA) using Dichotomous samplers (Partisan, 2000i-D, Thermo Scientific, Waltham, MA, USA) alongside SUPER SASS-Plus sequential speciation samplers (Met One Instruments, Inc., Grants Pass, OR, USA). The samplers were operated using a one-in-three or six-day schedule, with a 24-h sampling time (midnight–midnight) according to the protocol of the National Air Pollution Surveillance (NAPS) program. Details of the analytical methods are described elsewhere [42]. Briefly, PM_2.5_ and PM_10-2.5_ samples collected by the dichotomous sampler were subjected to the gravimetric determination of the PM mass and were subsequently analyzed for 22 elements using nondestructive X-ray fluorescence spectrometry (Epsilon 5 ED-XRF, Malvern Panalytical Inc., Longmont, CO, USA). PM_2.5_ samples were then analyzed for trace elements by inductively-coupled plasma mass spectrometry (ICP-MS; Agilent Technologies, Wilmington, DE, USA), combined with microwave-assisted acid digestion, which provides superior detectability for trace metal(oids) [48]. The analytical parameters for elemental analysis by both methods are summarized in Table S1. In order to better understand the contribution of road traffic to the fine and coarse particulate matter trace elements, a subset of weekday/weekend PM_10-2.5_ samples from each site and season was also analyzed by ICP-MS, following the same digestion and measurement method as the PM_2.5_ samples. For the determination of water-soluble elements, PM_2.5_ samples from the speciation sampler were extracted in 15 mL of deionized water (Milli-Q, > 18 MΩ, Millipore, Bedford, MA, USA) via sonication in a water bath for 30 min. The extracts were acidified by adding nitric acid (high-purity trace metal grade, 1% *w*/*v* final solution). The levels of water-soluble metals were quantified by ICP-MS. The method detection limits (MDLs) were determined based on the standard deviation of the measurements of laboratory blanks and low-level-concentrations spikes (Table S1). Field blank filters were analyzed together with samples for each of the methods. The average concentrations for all elements were below MDLs, except for Al and Zn in water-soluble extracts. For these elements, the average field blank concentration was used for blank corrections. The expanded relative uncertainty (ERU) was estimated using the historical QA/QC data generated during the analysis of routine test samples and duplicate measurements of samples.

### 2.3. Data Analysis and Processing

For the calculation of summary statistics, values below MDL were replaced by ½ MDL. For other statistical calculations, such as the nonparametric Kruskal-Wallis one-way analysis of variance (ANOVA), only the values above MDL were used. Grubb’s test (*p* < 0.05) was used for the identification and removal of outliers. Seasons were defined as warm (April to September), and cold (October to March). Statistical data analysis was performed using the STATISTICA ver.13 (StatSoft Inc., Tulsa, OK, USA) software.

The amount of crustal material (CM) in PM_2.5_ and PM_10-2.5_ was calculated based on the concentrations of the oxides of the major elements i.e.,: Al, Si, Ca, K, Ti and Fe, following a widely used approach [49] given by Equation (1)

CM = 1.89 ∙ [Al] + 2.14 ∙ [Si] + 1.4 ∙ [Ca] + 1.2 ∙ [K] + 1.7 ∙ [Ti] + 1.43 ∙ [Fe](1)

This particular empirical equation form is based on the assumption that Al, Si, Ca, K, Ti, Fe, and all elements associated with the earth’s crust, soil, and/or resuspended road dust, are mostly present as oxides. All other measured elements were classified as “trace elements”.

### 2.4. Positive Matrix Factorization Analysis

Positive matrix factorization analysis (PMF) is an advanced factor analysis technique based on a weighted least square fit approach [50]. It uses realistic error estimates to weight data values and imposes non-negativity constraints in the factor computational process. The identification and quantification of possible sources of FPM- and CPM-bound trace metals was accomplished by applying the elemental data to a multivariate receptor model using the EPA PMF5.0 software. This model has been widely employed in various studies and is described elsewhere [51,52]. In this work, PMF was performed on a small dataset obtained by pooling together 43 fine and 43 coarse samples (86 total) at each near-road site. A combined dataset with 24 variables was used as input to the model. The descriptive statistics of the elemental composition in the PMF are shown in Table S2. Running PMF on the two size fractions separately resulted in the model not functioning correctly. Therefore, the size of the dataset was increased by combining the FPM and CPM data at each near-road site, which is possible when the sources affecting both size fractions have similar profiles and temporal variations. This approach is not new and was also previously successfully applied to datasets with a limited number of samples, and to datasets with a limited number of species [53,54]. As reported by Scerri et al. [54], the use of the in-built rotational tools in the EPA PMF5.0 allowed to fully characterize the PMF solution for assessing the solution stability of relatively small datasets (<100 samples).

Input datasets were prepared according to the procedure by Norris et al. [55]. Data below the MDLs were set to MDLs/2, with an uncertainty of 5/6 of the corresponding MDL. Data > MDLs were used with uncertainty values equal to the ERU with the addition of 1/3 of the MDLs. The best solutions of PMF analysis were determined according to several criteria and guidelines [51,52]: (i) knowledge of the sources affecting the study area; (ii) the Q-value with respect to the expected (theoretical) value and its stability over multiple runs (*n* = 100); (iii) the number of absolute scaled residual > ±3; and (iv) finding profile uncertainties calculated by bootstrap (BS, *n* = 100), displacement (DISP), and bootstrap-displacement (BS-DISP) methods with an acceptable range. A more detailed description and justification of the PMF solution are presented in the Supplementary Material (Tables S3–S5; Figures S1 and S2).

## 3. Results and Discussion

### 3.1. PM Levels

Summary statistics for concentrations of PM fractions are presented in Table 2 and Table 3 for PM_2.5_ and PM_10-2.5_, respectively. As reported previously [42], the median PM_2.5_ mass concentrations were generally higher in both near-road sites as compared with nearby background urban locations. The Canadian Ambient Air Quality Standards (CAAQS) for PM_2.5_, calculated as the three-year average of the annual 98th percentile of the daily 24-h concentrations, is 27 μg m^−3^. The 98th percentile of the daily 24-h concentrations, calculated for every year at each site, ranged from about 14 μg m^−3^ to 26 μg m^−3^, and the three-year average did not exceed this limit at any of the NR sites included in this study. On average, PM_2.5_ constituted 49–52% of PM_10_ (FPM + CPM). Seasonal and weekday/weekend variations of fine and coarse PM fractions are presented in Figure S3. The PM_2.5_ and PM_10-2.5_ concentrations did not show any significant seasonality trend (Kruskal-Wallis test, *p* < 0.05). The weekday/weekend patterns of PM_2.5_ and PM_10-2.5_ concentrations at both near-road sites reveal that particle mass concentrations are strongly influenced by traffic emissions (Figure S3b). The relatively higher weekday/weekend enhancement for the PM_10-2.5_ fraction is consistent with the fact that CPM fraction is less easily transported in the atmosphere than FPM, resulting from the fact that local sources have a larger impact on the PM_10-2.5_ levels measured near roadways. For example, weekday/weekend mean increases of ~32% PM_2.5_ and ~42% of PM_10-2.5_ concentrations are found at the NR-TOR site.

### 3.2. Elemental Concentrations

Descriptive statistics for the elemental compositions of PM_2.5_ and PM_10-2.5_ collected over the study period are summarized in Table 2 and Table 3, respectively. Overall, the total metal concentrations in fine and coarse PM were higher at both near-road sites as compared to those observed at the nearby background urban sites. However, no metals or elemental species were found to exceed the Ambient Air Quality Criteria (24-h average) set by the Ontario Ministry of the Environment [56]. The most abundant elements at all sites were Al, Si, Ti, K, Ca, and Fe, with their sum accounting for an average of 95% of the total measured elements in both PM fractions. These elements exhibited a strong preference towards the coarse size mode, indicating that they largely originated from the crustal mineral component of road dust. In the coarse mode, crustal material contributions, determined by summing the oxides of crustal elements, were significant at both near-road locations (Figure S4), ranging on average between 28% and 32% of PM_10-2.5_. The higher concentration of mineral dust in coarse mode observed at NR-VAN is likely, in part, due to the closer proximity of the NR-VAN site to the roadside than that at the NR-TOR, resulting in a less diluted CPM at the Vancouver site. In the fine mode, the CM contribution to PM was lower, averaging ~6–8%. Averaged by season, the CM concentration in both PM fractions exhibited generally higher concentrations during the warmer months, probably due the combined effects of drier conditions and the absence of snow cover, especially in Toronto [42]. The increased concentrations of crustal elements in both fractions during the weekdays, as compared to weekends, demonstrates the significant effect of traffic in the dust resuspension process.

Among the crustal elements, PM_2.5_-bound Fe exhibited the highest enhancement at both near-road sites, indicating the significant contribution of the road traffic to the anthropogenic source of Fe. As reported previously, the local traffic contributed to about 70% and 60% of Fe in PM_2.5_ at the NR-TOR and NR-VAN sites, respectively [42]. Apparently, traffic activities related to brake wear and the resuspension of road dust, especially by heavy-duty vehicles, significantly contributed to the anthropogenic source of Fe at both studied sites. It has been proven that iron oxides typically represent the major component of the friction layer of braking systems [9,57] and are also dominant compounds in airborne and non-airborne wear debris [57,58,59]. Coarse iron enrichment, especially at the NR-VAN site, located at the major intersection on a heavily used trucking route, is likely a consequence of the iron-rich debris from heavy-duty trucks.

**Figure 2:**
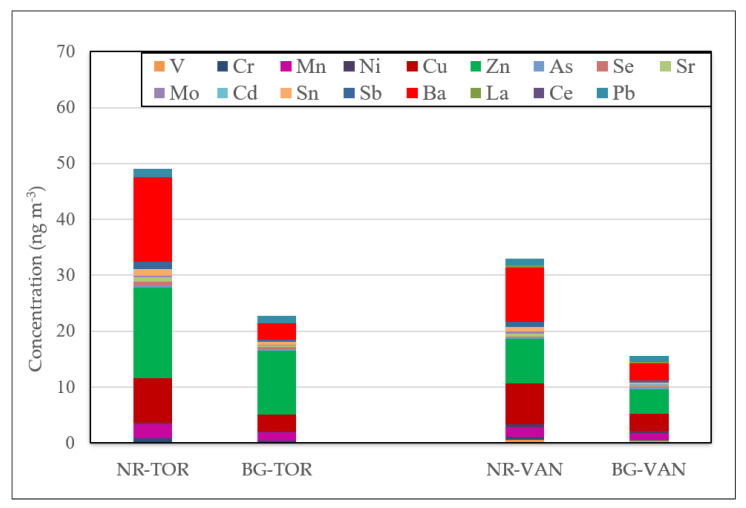
Median concentration of trace elements in PM_2.5_ samples collected over the study period listed in Table 1.

Consistent with other worldwide near-road studies, the concentrations of certain trace elements, such as Ba, Fe, Cu, Cr, Mo, Sb, and Sn in PM_2.5_, many of them classified as air toxics [60], were generally higher at both near-road sites than those at the nearby urban background locations (Figure 2 and Figure S5).

In particular, the greater enhancement of barium, iron, copper, and antimony than other elements, at both near-road sites, confirms that these metals are associated with non-exhaust emissions (e.g., abrasion of vehicle brake pads and discs/drums) [9,61,62]. It has been reported that heavy-duty vehicles (HDVs) are stronger emitters of Ba than low-duty vehicles (LDVs) because of the greater wear at each braking for an HDV than an LDV, as HDVs weigh more than LDVs [30,63,64,65]. These findings are confirmed by the higher median concentration of Ba on weekdays, especially at the Toronto highway site (Figure S6a), where the higher fraction of HDVs was observed [47,65]. On the other hand, weekday and weekend median concentrations of Cu and Sb did not vary significantly at both near-road sites, indicating a source with similar patterns [40,41]. Due to the larger variations in the chemical composition of brake material, the enhancement of other elements, such as Ti, Mo, and Sr, was also found (Figure S5). These metals have all been identified as constituents of vehicle brakes, although usage will depend upon the manufacturer and specification of the brake, and other sources of these metals that may be present [66].

The warm/cold season difference, especially observed in close proximity to the highway in Toronto, is likely due to increased traffic-induced road dust resuspension during the warm dry season (Figure S6b). On the other hand, the lower levels of metals observed in Vancouver during the warm, rather than the cold, season may be associated with the higher abundance of precipitation occurring in that area that could decrease metallic elements concentrations, directly via removal mechanisms. Meteorological parameters play an important role in regulating the concentration of metallic elements in PM_2.5_, especially for the removal efficiency of rainfall on those pollutants. This observation is in agreement with the results of recently reported studies in the Los Angeles area [43].

Other heavy trace elements, such as V, Ni, Se, As, Cd, and Pb showed a slightly different spatial distribution pattern, demonstrating the presence of other important sources of metals in urban PM_2.5_ (Table 2). For example, the concentrations of V and Ni, typical tracers of heavy oil combustion, measured at the Vancouver locations were almost three times higher than in Toronto, indicating a significant effect of marine transportation emissions in this area (situated close to the Port of Vancouver). Moreover, the elevated concentrations of La and La/Ce ratios > 2 at the Vancouver sites suggest the impact of marine transportation and/or the oil refining industry [67,68,69]. As shown in Figure S5, no significant enhancement of As, Se, Cd, and Pb was observed at both near-roadways, indicating their likely influence from transboundary pollution [41,70,71].

#### 3.2.1. Fine/Coarse Distribution of Elements

**Figure 3:**
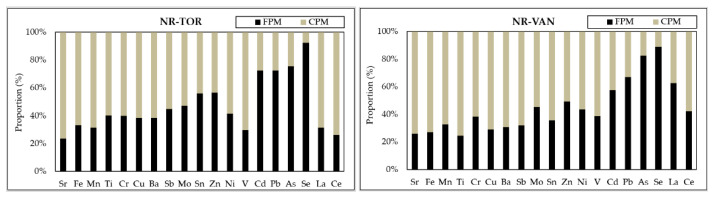
Relative contributions (%) of fine and coarse elements in PM_10_ at the near-road sites.

To illustrate how the size distribution of elements differs across near-road locations, a subset of PM_10-2.5_ samples from each site was also analyzed for trace elements by ICP-MS. As shown in Figure 3 and Table S2, the trace elements showed a clear partitioning trend between the PM_2.5_ and the PM_10-2.5_ fraction, thus reflecting the origin of some of these elements. As stated above, Fe is a major component of mineral dust, but can also be emitted from traffic-related activities, such as wear from braking and the resuspension of road dust [57], which explains its preferable coarse mode occurrence.

At the NR-TOR site, brake-wear-related elements (i.e., Ba, Cu, and Sb) are roughly evenly distributed between fine and coarse fractions, while they were mainly present in the coarse fraction at the NR-VAN site. According to Gietl et al. [61], PM originating from brake wear is the result of two processes. Mechanical wear (abrasive and fatigue wear) typically leads to the release of larger particles, belonging mainly to coarse or fine fractions, whereas brake pad materials contribute to the smaller particle size fractions by volatilization and condensation. For example, Cu, used as high-temperature lubricant in brake linings, is emitted primary from mechanical wear [64]. As reported by Bukowiecki et al. [72], brake-wear particles from light-duty vehicles were distributed in the entire size range larger than 1 μm, while the contribution from the submicrometer mode was very low. In contrast, more than 75% of the brake-wear emissions from heavy-duty vehicles were found in the coarse mode. Sternbeck et al. [63] reported that wear particles can accumulate on the wheel rims during braking and later be emitted by resuspension from the wheel rims. Thus, besides vehicle-type specific brake-wear emissions, road-dust resuspension is likely a dominant contributor of brake-wear elements. Higher levels of brake-wear metals in the coarse mode observed at the NR-VAN site is also, in part, due to the closer proximity of this site to the roadside as compared to the NR-TOR. Chromium was also mostly found in the coarse mode fractions. The elevated Cr concentrations in the coarse mode at near-road sites in Toronto and Vancouver indicate that local traffic activities, such as the abrasion of traffic infrastructure and asphalt surfaces, might have influenced the Cr concentrations at these stations [73]. Chromium has also been stated to derive from stainless steel and yellow road markings in traffic environments [74]. For Zn, used in rubber as ZnS and considered as tracer for tire wear [8], a similar pattern was observed at both traffic sites, its concentrations being nonetheless enhanced in PM_2.5_, due to its presence in additional anthropogenic emissions, including industrial- and exhaust-emitted aerosols [30,40].

Elements associated with combustion sources, such as fossil fuel and heavy-oil combustion (Ni, V), or high-temperature industrial processes (As, Cd, and Pb and Se), occur mostly in fine particles, although a coarse component was also visible. This indicates that traffic emissions did not significantly affect their concentrations during this study but, rather, their long-range transport. Other analyzed metals, such as La and Ce, were mostly found in the coarse mode in Toronto, whereas these elements had a mixed fine and coarse distribution in Vancouver. This may be linked to the anthropogenic sources of La and Ce in PM_2.5_ associated with marine transportation and/or oil refining industry emissions in the Vancouver area [67,69]. Overall, this variability in size may have an effect on the impact on human health, since the size of the particles greatly affects their transport and deposition in the respiratory tract and the lungs [75].

#### 3.2.2. Water-Soluble Metal(oids)

**Figure 4:**
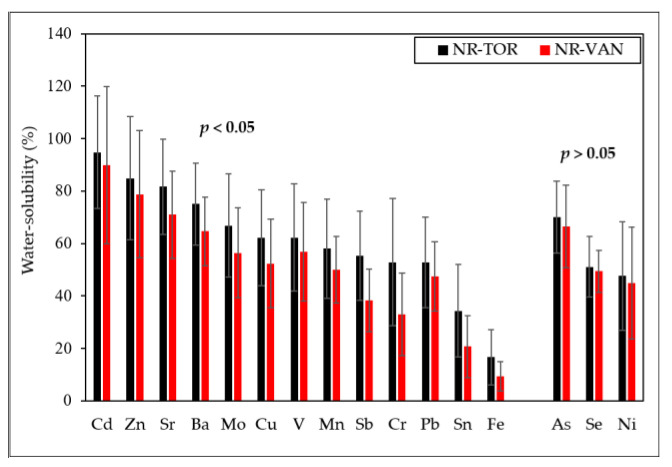
Mean water-solubility (%) of specific PM_2.5_-bound elements at near-road sites. Error bars represent standard deviation. Kolmogorov-Smirnov test with 95% probability was used to compare solubility of elements at two near-road sites.

Since a number of redox-active metals (i.e., Cu, Fe, Sb, Zn) were identified in elevated concentrations at both near-road environments, the water-solubility of measured elements in PM_2.5_ was also characterized. Figure 4 shows the average water-solubility of selected metal(oids), determined by the proportion (in %) of water-extracted metals concentration, and those from strong acid digestion.

Overall, the percent of water-solubility of most of the trace elements for both near-road sites are comparable, and similar to other studies [39]. The most water-soluble metals were Cd and Zn (>80%), and the least soluble was Fe (<20%). Metals primarily originating from brake wear, such as Cu, Ba, and Sb, have a mean solubility that varied from ~40 to ~70% at both sites. This behavior can be due to the diverse sources of metals, such as metal-dominated abrasion or hot-vapor condensation particles, or metals that have condensed onto the surface of other particles and, thus, tend to be more labile than metals bound within crustal material. For example, a relatively high solubility of fine Zn is likely attributed to its high-temperature industrial sources (See Section 3.3). While Fe exhibited a low solubility, it is the most abundant soluble metal, followed by Zn, Ba, Cu, Mn, and Sb. This is important, as water-soluble Fe and other transition metals, specifically metals associated with brake/tire wear (i.e., copper), have been directly correlated to ROS production [24,28]. Furthermore, other studies also reported associations between ROS activity and water-soluble brake wear elements (Cu, Ba) in coarse particles [23,46].

A number of these metal(oids) are typically in the form of oxides or other compounds and, thus, have varying solubility depending on their specific form. As demonstrated in Figure 4, slightly lower water-solubility was observed for brake-wear elements in the PM_2.5_ collected in Vancouver. For example, the lower water-solubility of PM_2.5_-bound Sb might be due to the presence of a mixture of Sb(III) and Sb(V) oxides in different relative abundances and, thus, solubility, as compared to the NR-TOR site. According to Verrica et al. [76], antimony, present in brake pads as Sb_2_S_3_, is oxidized during the brake abrasion process to more stable compounds, such as antimony-mixed oxide forms, and apparently their ratio does not change according to any specific rule. Thus, the higher solubility of Sb observed in Toronto is likely a result of PM_2.5_ containing Sb(V), primarily (Figure 4). It is worth noting that Sb_2_O_3_ is a compound that is categorized as a possibly carcinogenic substance and has been shown not to be soluble in water, but partially soluble in physiological fluids and, therefore, special attention is drawn to its atmospheric concentration levels [76].

### 3.3. Source Identification and Apportionment of Trace Elements in Fine and Coarse PM

**Figure 5:**
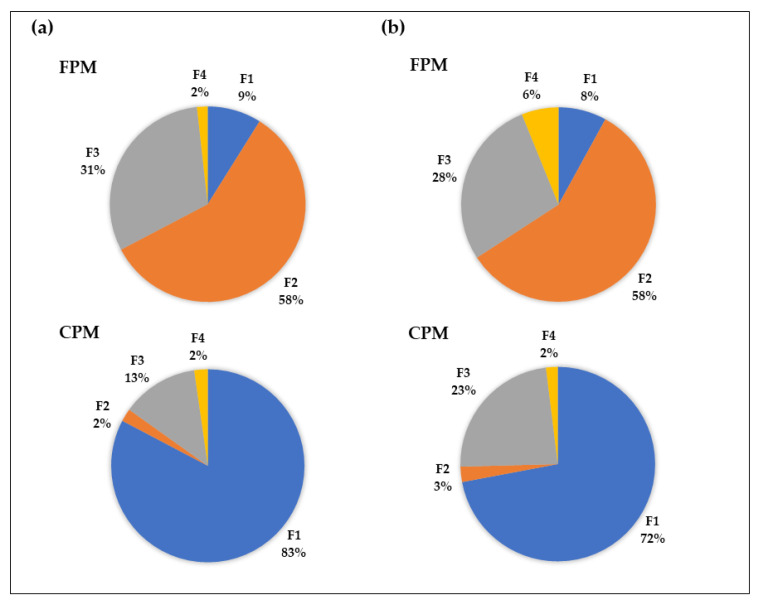
Relative contribution (%) of PMF-resolved factors/sources to the total element concentration in fine and coarse PM samples collected at (**a**) NR-TOR and (**b**) NR-VAN. F1 = Mineral/Road Dust; F2 = Regional/Local Industry; F3 = Brake/Tire Wear; F4 = Unexplained.

As mentioned earlier, a combined set of fine and coarse samples (86 samples total) analyzed for 24 elements at each site was used as input to the EPA PMF5.0 model. This approach has proven to increase the statistical significance of the analysis, although it assumes that the elemental profiles of the sources do not vary at two PM fractions. The best solution was obtained for four factors (Figure S7). The modeled time series of the contributions (ng/m^3^) from each source are shown in Figure S8. The results for the average contribution of identified sources/factors to the total concentrations of elements in the PM_2.5_ and PM_10-2.5_ fractions at both the near-road sites are shown in Figure 5.

#### 3.3.1. Factor 1: Non-Exhaust: Mineral/Road Dust

The first source appears to represent resuspended mineral material and road dust; therefore, it was labelled “mineral/road dust”. It is characterized by strong contributions from the major crustal elements, such as Si, Al, Ca, Fe, Mn, and K, and elements associated with exhaust emissions from burnt lubricating oil (Ca, Zn), as well as brake and tire wear (i.e., Cu, Ba, Sb, Fe, Zn), and traffic infrastructure/asphalt surfaces (Cr). According to Figure 5, this non-exhaust emission source is by far the major contributor to the total element concentrations of coarse PM, accounting for 72% (1.18 μg m^−3^) and 83% (1.67 μg m^−3^), respectively, at NR-VAN and NR-TOR. The mineral/road dust factor makes the third largest contribution (8–9%) to the total PM_2.5_ element concentrations at both sites. Apparently, turbulence induced by vehicular movement (especially heavy-duty vehicles) was a major resuspension source of particles already present on the road surface. Resuspended particles, therefore, comprise particles from non-exhaust sources (brake wear, tire wear, road-surface wear), as well as particles from other sources that have also deposited onto the road surface, for example, exhaust emission particles, particles from de-icing and gritting, and wind-blown dust particles [9,11].

#### 3.3.2. Factor 2: Regional/Local Industry

The second source contributes the largest fraction (~58%) to the total PM_2.5_ elemental concentrations at NR-TOR (~0.51 μg m^−3^) and NR-VAN (~0.34 μg m^−3^) and is the major contributor to the concentrations of S, Se, As, K, Cd, and Pb (Figure S7). These elements have commonly been distinguished as tracers for coal combustion or coking emissions, which suggests that this factor can be attributed to the long-range transport of coal-fired power plant emissions. This is consistent with previous studies [27,40,70,71]. This factor may also include a multitude of smaller nearby industrial sources that might not be specifically distinguished by the model, but apparently exist in the area; for example, the high loading of Zn and Mn in this factor in Toronto could be from local stationary industrial sources, such as metals processing [40].

#### 3.3.3. Factor 3: Non-Exhaust: Brake/Tire Wear

Factor 3 appears to be the second non-exhaust emission source characterized by high loads of Ba and Cu, in addition to other elements (Fe, Sb, Sn, Mo, and Sr), commonly associated with the abrasion of brake pads and discs/drums [9,57,61,62]. The presence of Zn in this factor may imply that this source can also be associated with tire wear [8]. Other elements, such as Cd, Cu, and Pb are also utilized in tire manufacturing and, thus, may be emitted from tire-pavement abrasive emissions [8]. Overall, brake/tire wear sources accounted for a considerable portion of the coarse and fine elemental mass at both sites (Figure 5). This source explained about 31% (0.27 μg m^−3^) and 28% (0.17 μg m^−3^) of PM_2.5_ element concentrations at the NR-TOR and NR-VAN sites, respectively. It shows the greater contribution to the total PM_10-2.5_ element concentrations in Vancouver (23%; 0.38 μg m^−3^) than in Toronto (13%; 0.26 μg m^−3^). This suggests that frequent stop-and-go driving, and the low speed of the vehicles at the major intersection on a heavily used trucking route in Vancouver, in addition to less dilution compared to the NR-TOR, may, in part, result in the greater emissions of mechanically generated brake/tire wear and, thus, greater contributions to the PM_10-2.5_ element concentrations [7,58]. The compositional differences may also affect the rate of particle emissions [77] and, most probably, the physical properties of emissions.

#### 3.3.4. Factor 4: Unexplained

As a minor source, this factor was dominated by the presence of La and Ce, suggesting that the source originates from automotive catalysts and/or fluidized catalysts used in refining [67,68,69,78,79]. In recent years, however, the engineered nanoparticles of Ce (CeO2) have been increasingly used in other traffic-related applications, such as a catalyst in diesel vehicles, to reduce particulate emissions [80,81]. The interpretation of the fourth factor in Toronto is not as clear. However, the presence of elements associated with exhaust emissions (i.e., V, Ni, Cu, Ba), in addition to La and Ce, suggests vehicle emissions [30,80]. Overall, this factor accounted for a small percentage (~2%) to the total elemental concentrations for both size PM fractions, and so does not represent a significant source. In Vancouver, the fourth factor also shows a high content of vanadium, a typical tracer for many oil combustion sources, such as oil-fired power plants, ships, and ferries, as well as homes and commercial buildings (for heating). Since NR-VAN is within 3 km of the Port of Vancouver, and less than 9 km from a petrochemical/refining complex, this source is considered as refining/marine transportation. This factor accounted for ~6% of PM_2.5_ element concentrations at this site.

**Figure 6:**
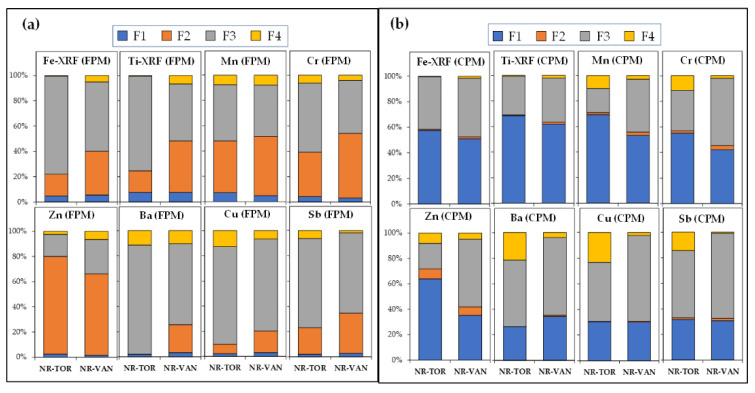
The relative contribution of PMF-resolved sources to individual redox-active metals in (**a**) PM_2.5_ (FPM), and (**b**) PM_10-2.5_ (CPM). F1 = Mineral/Road Dust; F2 = Regional/Local Industry; F3 = Brake/Tire Wear; F4 = Unexplained.

Figure 6 and Figure S9 illustrate the relative and absolute contribution of PMF-resolved sources to the concentrations of individual redox-active metals, respectively. As shown at these figures, the non-exhaust emission from road traffic was the major contributor to the various redox-active metal species at both near-road sites for this period. As an example, ~78% (6.6 ng m^−3^) and ~74% (7.4 ng m^−3^) of fine Cu were estimated from brake-wear emissions at NR-TOR and NR-VAN, respectively. In general, the greater estimated contribution of this source to the fine redox-active metals was observed at the busy Toronto highway site, where it can be assumed that the heavy metal concentrations were greatly affected by fleet composition and volume, as well as the different types of braking systems (i.e., disk brake vs. drum brake, low-metallic vs. semi-metallic, driving behavior and regulations) [57].

The brake/tire source also contributed significantly to Cu and other metals in coarse PM, especially in Vancouver. Furthermore, the absolute contribution of brake/tire source to the total Cu concentration in PM_10_ was estimated to be 13.0 ng m^−3^and 24.4 ng m^−3^ at NR-TOR and NR-VAN, respectively. It is worth noting that copper usage in brake pad formulations has recently become the subject of considerable debate, primarily because of the potentially toxic effects of Cu and other heavy metals. As a result, brake pads manufactured after January 2021 must not contain more than 5% copper by weight [82].

As illustrated in Figure 6 and Figure S9, the resuspended road dust, presumably contaminated by heavy metals produced by vehicular exhaust, brake/tire wear, and road abrasion, was the dominant contributor to these CPM elements, especially Fe, Ti, Mn, and Cr, at both near-road sites. The observed higher relative contribution of this source, for example, to Zn in the CPM fraction at the Toronto highway site, is most likely attributed to the dominant impact of tire wear, as well as abrasion from the highway surface (asphalt).

## 4. Conclusions

In this study, the trace elemental composition of the fine and coarse fractions of airborne particles was analyzed at dense traffic areas and nearby urban background sites in Toronto and Vancouver. The most abundant elements (Al, Si, Ti, K, Ca, and Fe) exhibited a strong preference to the coarse-size mode, indicating that they largely originated from the crustal mineral component of road dust. Roadside enrichment was observed for a large number of elements associated with traffic emissions (i.e., Ba, Fe, Sr, Sb, Cu, Ti, Mn), while those elements that are typically from more regional sources (i.e., S, As, Se, Cd) were not found to have substantial increments. In particular, the greater enhancement of brake-wear-related elements (i.e., Ba, Cu, Fe and Sb), detected in the coarse mode as well, confirms that these metals are associated with the abrasion of brake pads and discs/drums. The results show that these elements are roughly evenly distributed between FPM and CPM at NR-TOR, while they were mainly present in the coarse fraction at the NR-VAN site.

Application of PMF to the combined fine and coarse metal data (86 total), with 24 observations at each site, allowed us to distinguish four main sources of trace metals in fine and coarse PM. This included traffic non-exhaust (crustal/road dust, brake/tire wear) sources, and regional and local industrial sources. Although there are constraints on the number of samples analyzed, it can be concluded that scientifically meaningful source apportionments were obtained by running PMF on datasets consisting of a small number of samples (less than the currently accepted rule of thumb) [53,54]. A resuspended crustal/road dust factor is the largest contribution (~72–83%) to the CPM trace element (i.e., Fe, Ba, Cu, Zn, Sb, Cr) concentrations produced by vehicular exhaust and non-exhaust traffic-related processes, that have deposited onto the surface. The second non-exhaust factor related to brake/tire wear abrasion accounted for a considerable portion of the fine and coarse elemental mass at both sites. This factor was estimated to contribute from ~28% of FPM element concentrations at NR-VAN, to ~31% at NR-TOR. A higher contribution of this factor to the CPM element concentrations was observed at NR-VAN (~23%) than at NR-TOR (~13%).

The results show that non-exhaust traffic-related processes were major contributors to the various redox-active metal species (i.e., Fe, Cu) in both PM fractions. A substantial proportion of these metals were water-soluble, which is an important contributor to the formation of reactive oxygen species and, thus, may lead to cell oxidative damage. Regional and local industry contributed mostly to the FPM elemental concentrations at both near-road sites. In addition, a distinct source factor was identified at NR-VAN in having ~6% (~40 ng m^−3^) contribution to fine-metal concentrations from a factor distinguished by La, Ce, and V, which appears to be associated with heavy oil combustion (marine transportation) and the oil refining industry.

In agreement with other studies [9,10,83,84], it becomes obvious that the level of elements, including some toxic and redox-active metals, depends on traffic intensity, the vehicle-fleet characteristics (i.e., heavy- vs. light-duty vehicles) and speed, the type of road surface, and the concentration of metals in the nearby background urban sites. As a result, the non-exhaust-based particles, and particles from other sources, create complex elemental PM mixtures at near-traffic sites. This information can ultimately aid in developing and implementing more targeted regulation to reduce exposure to traffic-generated trace metals in urban areas. Given that the vast majority of all road transport PM emissions will come from non-exhaust sources in the coming years because of the widespread penetration of electric vehicles, one of the most promising technological solutions for reducing exhaust emissions, more attention should now be given to non-exhaust PM emissions.

## Figures and Tables

**Table 1:** Description of the sampling locations and measurement period.

Site (NAPS ID)	Site Name	Latitude, Longitude	Site Type/Traffic Density (Veh/Day)	Measurement Period
Toronto, ON (060438) ^a^	NR-TOR	43.711, −79.543	Highway Open Terrain (365,000–411,600)	29 July 2015– 30 June 2017
Toronto, ON (060440) ^a^	BG-TOR	43.781, −79.467	Urban Background (traffic not allowed)	18 May 2015–27 April 2017
Vancouver, BC (100141) ^a,c^	NR-VAN	49.260, −123.078	Urban Road Street Canyon (~30,000)	11 July 2015– 30 June 2017
Vancouver, BC (100142) ^b^	BG-VAN	49.253, −123.049	Urban Background (traffic not allowed)	11 July 2015– 28 August 2016

^a^ 1 in 3 days; ^b^ 1 in 6 days; ^c^ 1 in 6 days (since 3 September 2016).

**Table 2:** Summary statistics for PM_2.5_ (in µg m^−3^) and elemental concentrations (in ng m^−3^) at Toronto and Vancouver sites.

	NR-TOR (*n* = 229)				BG-TOR (*n* = 113)				NR-VAN (*n* = 186)				BG-VAN (*n* = 70)			
	Mean	Median	S.D.	Max.	Mean	Median	S.D.	Max.	Mean	Median	S.D.	Max.	Mean	Median	S.D.	Max.
PM_2.5_	8.2	7.2	4.3	28.7	6.2	5.2	4.1	27.1	6.6	5.7	3.3	20.7	4.6	4.1	2.4	14.4
Al-XRF	21	8	20	95	19	8	17	81	22	9	22	105	18	11	19	112
Si-XRF	56	49	37	197	35	26	33	186	41	33	30	170	31	24	28	178
K-XRF	50	42	49	592	39	32	25	135	39	29	42	416	38	26	59	462
Ca-XRF	89	76	55	289	48	42	32	153	35	31	20	156	26	22	21	163
Ti-XRF	7	7	4	18	3.6	3.2	3	10	5.6	4.9	3	17	2.8	2.7	2	9
Fe-XRF	174	140	116	517	61	46	52	256	131	109	83	427	56	39	45	238
V	0.2	0.1	0.1	0.6	0.12	0.09	0.1	0.6	0.71	0.53	0.7	4.3	0.54	0.38	0.7	4.6
Cr	0.8	0.7	0.4	2.9	0.43	0.37	0.3	1.7	0.71	0.6	0.4	2.5	0.4	0.19	0.6	4.3
Mn	3.2	2.8	2.2	15	1.9	1.5	1.6	8.9	2.1	1.7	1.5	10.7	1.5	1.2	1.2	6.4
Ni	0.3	0.3	0.2	1.1	0.21	0.09	0.2	0.7	0.59	0.47	0.4	3.4	0.5	0.4	0.5	2.5
Cu	8.9	8	5.6	26.9	3.6	3	2	11.7	9	7.4	6	33.9	4.2	3.1	3.6	18.5
Zn	23.1	16.2	23	155	24.7	11.4	24	142	9.9	7.9	6	31	6.3	4.4	5	27
As	0.55	0.45	0.4	2.3	0.45	0.32	0.4	1.6	0.45	0.34	0.5	3.8	0.38	0.31	0.3	1.7
Se	0.71	0.57	0.8	7.5	0.58	0.37	0.5	3	0.15	0.14	0.1	0.5	0.14	0.13	0.1	0.3
Sr	1.19	0.8	4.4	56.4	0.39	0.3	0.4	3	0.64	0.49	0.9	10.5	0.51	0.14	1.6	12.8
Mo	0.35	0.31	0.2	1.38	0.18	0.15	0.15	0.74	0.44	0.38	0.26	1.44	0.22	0.18	0.15	0.64
Cd	0.09	0.06	0.34	4.49	0.09	0.05	0.34	2.96	0.06	0.04	0.07	0.5	0.08	0.04	0.12	0.7
Sn	1.32	1.15	0.8	4.4	0.71	0.52	0.6	3.3	0.86	0.71	0.6	3.5	0.41	0.31	0.4	2
Sb	1.45	1.32	0.84	4.85	0.55	0.41	0.54	4.08	1.17	0.98	0.72	4.08	0.56	0.47	0.38	1.82
Ba	17.6	15	13.1	57.4	3.6	2.8	2.7	13.6	11.4	9.7	6.8	40.5	4.3	3	5.5	41.9
La	0.04	0.03	0.02	0.14	0.03	0.02	0.03	0.17	0.59	0.35	0.66	3.23	0.41	0.27	0.48	2.68
Ce	0.06	0.06	0.03	0.18	0.03	0.02	0.02	0.13	0.19	0.11	0.23	1.9	0.11	0.06	0.15	0.72
Pb	1.9	1.5	1.5	9.4	2	1.3	1.3	7.1	1.5	1.1	1.3	9.8	1.6	0.97	2.4	17.9
Total	456	397	245	1219	245	214	153	755	315	264	171	967	194	155	125	699

**Table 3:** Summary statistics for PM_10-2.5_ (in µg m^−3^) and elemental concentrations (in ng m^−3^) at Toronto and Vancouver sites.

	NR-TOR (*n* = 229)				BG-TOR (*n* = 113)				NR-VAN (*n* = 186)				BG-VAN (*n* = 70)			
	Mean	Median	S.D.	Max.	Mean	Median	S.D.	Max.	Mean	Median	S.D.	Max.	Mean	Median	S.D.	Max.
PM_10-2.5_	8.7	5.9	9.6	73.5	5.9	4.9	3.9	21.0	6.4	5.5	4.0	32.2	4.5	4.6	2.0	9.4
Al-XRF	153	110	154	823	138	100	123	618	191	148	167	1000	108	70	94	308
Si-XRF	388	302	359	2270	340	222	300	1637	438	317	346	2200	264	219	188	695
K-XRF	47	37	37	221	44	39	31	165	43	38	24	153	37	35	18	109
Ca-XRF	488	346	454	2280	404	311	319	1501	149	120	107	607	108	88	69	333
Ti-XRF	14	11	11	52	11	8	8	35	17	15	10	56	8	7	5	25
Mn-XRF	5	3	4	17	3	2	3	10	4	3	3	14	2	1	1	6
Fe-XRF	297	221	237	1040	144	111	109	451	383	330	230	1242	156	128	113	591
Zn_XRF	11	8	9	50	12	6	20	104	9	7	6	40	4	3	3	14
Total	1383	942	1221	6067	971	710	849	4331	1233	995	948	5680	687	534	477	1836

## Data Availability

The data presented in this study are available in the article or in the Supplementary Materials.

## Supplementary Materials

The following are available online at https://www.mdpi.com/article/10.3390/toxics9100264/s1. Quality assurance and quality control of data. Table S1: Summary of analytical parameters of elemental analysis. S2. Positive matrix factorization analysis: A more detailed description and justification of the PMF solution used. Table S2: Summary statistics of elemental concentrations (in ng m^−3^) in a subset of PM_2.5_ and PM_10-2.5_ samples collected in Toronto and Vancouver. Table S3: Summary of diagnostics for PMF with 4 factors for NR-TOR data. Table S4: Summary of diagnostics for PMF with 4 factors for NR-VAN data. Table S5. Standard deviations (S.D.) of the Q-values (*n* = 100) for NR-TOR and NR VAN. Figure S1: Inflection curves of the Q/Q_expected_ value used to obtain the optimum number of PMF factors for (a) NR-TOR and (b) NR-VAN. An inflection at the 4-factor solution indicates that it is the most probable solution. Figure S2: Determination of potential optimal number of factors by using the maximum individual mean and standard deviation for (a) NR-TOR and (b) NR-VAN. Figure S3: Median concentrations of PM_2.5_ and PM_10-2.5_ in (a) seasonal and (b) weekday/weekend collected samples over the Study Period listed in Table 1 (Main text). Whiskers represent IOR. Figure S4: Contribution (in %) of crustal element oxides in PM_2.5_ and PM_10-2.5_ samples collected over the Study Period listed in Table 1 (Main text). Figure S5: Median ratios (NR vs BG) of element concentrations. Kolmogorov-Smirnov test was used to compare concentrations of elements measured simultaneously at the near road and nearby background sites in Toronto and Vancouver. Figure S6: Median concentrations of brake-wear elements in (a) weekday/weekend and (b) seasonal PM_2.5_ samples collected over the Study Period listed in Table 1 (Main text). Figure S7: Source profiles of the PMF identified factors for a subset of fine and coarse PM samples analyzed for trace elements by ICP-MS: (a) NR-TOR and (b) NR-VAN. F1 = Mineral/Road Dust; F2 = Regional/Local Industry; F3 = Brake/Tire Wear; F4 = Unexplained. Figure S8: Time series of PMF resolved source factor contributions of metals in CPM and FPM for (a) NR-TOR and (b) NR-VAN. Figure S9: The absolute contribution of PMF-resolved sources to the concentration of individual redox-active metals in (a) PM_2.5_ (FPM) and (b) PM_10-2.5_ (CPM). F1 = Mineral/Road Dust; F2 = Regional/Local Industry; F3 = Brake/Tire Wear; F4 = Unexplained. S3. References.
