# Supplementary material for: Insights into Elemental Composition and Sources of Fine and Coarse Particulate Matter in Dense Traffic Areas in Toronto and Vancouver, Canada
Source: Toxics. 2021 Oct 14;9(10):264. doi: 10.3390/toxics9100264 (PMC8537750; doi:10.3390/toxics9100264)
Supplement: Supplementary file 1 [file toxics-09-00264-s001.zip › toxics-1403005-supplementary.pdf]

toxics MDPI

Supplementary Materials: Insights into Elemental Composition and Sources of Fine and Coarse Particulate Matter in Dense Traffic Areas in Toronto and Vancouver, Canada

Valbona Celo, Mahmoud M. Yassine, Ewa Dabek-Zlotorzynska*

S1. Quality assurance and quality control of data

Sample analyses were performed in ISO-17025 accredited laboratories and were associated with analysis of a number of quality control samples, such as method blanks and spikes, duplicate measurements, and standard reference materials (SRMs). Expanded relative uncertainties (ERU) ranged from 5 to 25% and were usually higher for the ED-XRF analysis. Method detection limits (MDLs) were determined based on the standard deviation of measurements of laboratory blanks and low-level-concentrations spikes (Table S1).

Table S1. Summary of analytical parameters of elemental analysis.

Element	ICPMS (Acid Digested)		ICPMS (Water-Soluble)		ED-XRF	
	MDL (μg m⁻³)	ERU (%)	MDL (μg m⁻³)	ERU (%)	MDL (μg m⁻³)	ERU (%)
Be	0.01	10	0.01	10	NA	NA
Al	3.70	7	1.63	12	13.07	17
Si	NA	NA	NA	NA	5.34	15
S	NA	NA	NA	NA	1.03	10
K	NA	NA	NA	NA	5.34	5
Ca	NA	NA	NA	NA	2.21	7
Ti	0.19	10	0.27	9	1.41	11
V	0.07	5	0.02	7	0.57	11
Cr	0.37	8	0.33	6	1.07	18
Mn	0.06	6	0.10	7	2.25	14
Fe	6.48	5	1.63	9	5.34	6
Co	0.02	9	0.02	7	NA	NA
Ni	0.19	8	0.08	8	0.85	15
Cu	0.28	8	0.68	8	NA	NA
Zn	0.93	8	1.04	9	1.67	11
As	0.03	6	0.04	10	NA	NA
Se	0.05	9	0.09	9	1.71	5
Br	NA	NA	NA	NA	1.92	20
Sr	0.28	7	0.05	6	4.26	5
Mo	0.02	8	0.09	9	NA	NA
Ag	0.01	10	0.04	8	NA	NA
Cd	0.02	8	0.03	10	5.34	10
Sn	0.09	5	0.07	6	8.48	1
Sb	0.02	6	0.04	6	5.64	3
Ba	0.19	6	0.10	7	18.91	17
La	0.002	10	NA	NA	NA	NA
Ce	0.002	9	NA	NA	NA	NA
Tl	0.03	8	0.01	8	NA	NA
Pb	0.09	8	0.21	7	5.34	24
U	0.01	8	0.01	9	NA	NA

NA= not analyzed; ERU = Expanded relative uncertainty (%).

S2. Positive Matrix Factorization Analysis

Positive matrix factorization (PMF), a receptor-based source apportionment model, is based on a least squares method [1–3]. A detailed description of PMF analysis are presented by Norris et al. [4]. Briefly, the source apportionment problem is solved by PMF through the decomposition of x_{ij}, the matrix made up of the *j* chemical species analysed in the characterisation of the *i* (daily) samples into the f_{kj}, the matrix representing the chemical composition of each of the *p* element sources and the g_{ik}, the matrix representing the daily contributions of each source, which is defined as:

$$x_{ij} = \sum_{k=1}^{p} g_{ik} f_{kj} + e_{ij} \quad (1)$$

in which e_{ij} is the matrix of residuals. The goal of multivariate receptor modeling is to determine the number of sources (*p*), the source contributions (g_{ik}), and the chemical profiles (f_{kj}) of identified sources. Factor contributions and profiles are derived by minimizing the object function (Q), which is defined as:

$$Q = \sum_{i=1}^{n} \sum_{j=1}^{m} \left[\frac{x_{ij} - \sum_{k=1}^{p} g_{ik} f_{kj}}{u_{ij}} \right]^2 \quad (2)$$

where u_{ij} is the uncertainty in the jth element for the ith sample. This factor analysis ensures that all of the species profiles (matrix *F*) should be non-negative and each sample must have a non-negative source contribution (matrix *G*). PMF is able to simultaneously change the elements of *G* and *F* in each iterative step so that Q is minimized.

Other PMF parameters such as residual analysis including maximum individual mean (IM) and maximum individual standard deviation (IS), G-space plots, and Fpeak rotational analysis can be employed to reduce the number of meaningful factor solution. The scaled residuals were used to detect data anomalies (deviated from the normal distribution). For instance, if the input data and the model are correct, the plot of the scaled residual values against their occurrences would show a random distribution with the majority of them located between −2 and +2 [4]. Moreover, the information from the scaled residual matrix (R) in the PMF is useful tool to reduce the ambiguity in choosing the range of the meaningful number of factors. Each element in the matrix R is determined by [5]:

$$r_{ij} = \frac{e_{ij}}{u_{ij}} \quad (3)$$

For each specific number of factors, two parameters can be obtained from the scaled residual matrix, including maximum individual mean (IM) and maximum individual standard deviation (IS):

$$\mathrm{IM} = \max_{j=1\ldots m} \left(\frac{1}{n} \sum_{i=1}^{n} r_{ij} \right) \quad (4)$$

$$\mathrm{IS} = \max_{j=1\ldots m} \left(\frac{1}{n-1} \sum_{i=1}^{n} (r_{ij} - \bar{r}_j)^2 \right) \quad (5)$$

IM and IS are used as indicators to identify species that has the least and the most impricise fit respectively. When the number of factors increases to a critical value, both IM and IS will experience a drastic drop in their values and thus indicate the most probable optimal PMF solution.

The optimal number of factors can be also assessed by the examination of G-space plots for the proposed optimal PMF solution. G-space plot, which is simply a scatter plot between one factor *vs* another, can be helpful to indicate the relationship between source contributions and rotational ambiguity. A combination of factors with no points on or near the axes results in greater rotational ambiguity. In addition, Fpeak is a parameter used to explore the rotational ambiguity of PMF solution by examining the Q-value due to Fpeak rotation as well as the corresponding G-space plots of the Fpeak solution factor to see if the points move toward axes.

2.1. Data Preparation

Data preparation is one of the most critical processes for the PMF analysis. Descriptive statistics and temporal variations of metal data were examined to verify the quality of data set, including understanding of outliers for each species. Data validation tests to identify values that appeared abnormal as compared to the overall data were carefully performed using scatter plot and time series analysis.

In this work, PMF was performed on a small dataset obtained by pooling together 43 fine and 43 coarse samples (86 total) at each near-road site. A combined dataset with 24 variables was used as input to the model using the EPA PMF5.0 software. Major elements (i.e., Al, Si, K, Ca, Ti, Fe and S) detected by ED-XRF method were chosen, while other trace elements (i.e., Cu, V, Ba, Zn, As, Se, Cd, Pb) detected in both the fine and coarse PM fractions were obtained from the near-total ICP-MS analysis du to the higher sensitivity for ICP-MS. Summary of the input datasets for the PMF analysis are presented in Table S2.

Table S2. Summary statistics of elemental concentrations (in ng m^{-3}) in a subset of $PM_{2.5}$ and $PM_{10-2.5}$ samples collected in Toronto and Vancouver.

	**$PM_{2.5}$**								**$PM_{10-2.5}$**							
	**NR-TOR (*n* = 43)**				**NR-VAN (*n* = 43)**				**NR-TOR (*n* = 43)**				**NR-VAN (*n* = 43)**			
	Mean	Median	S.D.	Max	Mean	Median	S.D.	Max	Mean	Median	S.D.	Max	Mean	Median	S.D.	Max
Al-XRF	19	5.4	22	91	22	15	21	88	241	198	175	785	252	201	169	637
Si-XRF	70	57	50	197	52	47	32	163	571	419	414	2000	572	483	356	1411
S-XRF	455	344	362	1914	236	218	110	546	58	53	36	178	53	48	22	132
K-XRF	57	47	30	169	60	43	65	416	68	53	43	207	56	51	21	105
Ca-XRF	106	91	66	289	43	39	18	94	718	620	472	2052	195	159	113	429
Ti-XRF	8.0	7.5	4.4	17	7.0	5.9	3.2	17	19	17	12	52	22	19	10	55
Fe-XRF	218	193	121	505	182	158	92	456	388	316	239	921	503	448	253	1242
V	0.25	0.34	0.12	0.43	0.62	0.35	0.50	2.2	0.35	0.27	0.24	1.1	0.82	0.67	0.46	1.9
Cr	0.85	0.69	0.38	2.3	0.93	0.69	0.60	2.9	1.3	1.2	0.76	3.3	1.8	1.4	1.2	5.9
Mn	3.4	2.5	2.4	12	2.5	2.2	1.4	6.4	6.5	5.5	4.1	20	5.1	4.4	2.5	12
Ni	0.52	0.68	0.22	0.78	0.72	0.69	0.32	1.9	0.58	0.39	0.33	1.4	1.0	0.75	0.68	3.2
Cu	8.3	7.2	5.2	19	9.7	8.5	5.8	30	14	12	8.9	34	27	21	16	88
Zn	28	21	25	102	11	10	7.0	39	17	16	10	54	12	11	6.2	37
As	0.53	0.35	0.47	2.3	0.49	0.42	0.32	2.0	0.12	0.09	0.07	0.34	0.10	0.07	0.06	0.33
Se	0.47	0.37	0.39	2.0	0.15	0.14	0.04	0.28	0.05	0.07	0.03	0.12	0.05	0.05	0.03	0.07
Sr	0.95	0.73	0.72	4.0	0.68	0.56	0.64	4.4	2.8	2.4	1.6	6.6	1.8	1.6	0.75	3.7
Mo	0.34	0.31	0.18	0.87	0.52	0.47	0.25	1.2	0.38	0.35	0.20	0.82	0.75	0.57	0.40	1.79
Cd	0.07	0.06	0.05	0.27	0.06	0.05	0.06	0.35	0.03	0.02	0.02	0.15	0.06	0.03	0.10	0.47
Sn	1.6	1.5	1.1	5.0	1.3	1.0	0.90	5.3	1.3	1.2	0.76	2.7	2.3	1.8	1.4	6.5
Sb	1.4	1.4	0.79	3.0	1.3	1.2	0.69	3.6	2.0	1.7	1.3	4.6	3.2	2.5	1.7	8.1
Ba	17	13	13	45	12	11	7.1	35	27	21	19	76	29	24	14	77
La	0.02	0.02	0.03	0.13	0.37	0.08	0.58	2.5	0.06	0.06	0.07	0.38	0.21	0.07	0.31	1.1
Ce	0.04	0.03	0.04	0.16	0.13	0.05	0.20	0.74	0.12	0.10	0.15	0.76	0.16	0.07	0.22	0.73
Pb	1.8	1.5	1.3	7.4	1.6	1.4	0.9	4.5	0.7	0.6	0.5	2.4	0.9	0.7	0.9	5.0

Input variables for the PMF analysis were carefully selected based on the percentages of missing and below the method detection limit (MDL) as well as the estimate of signal-to-noise ratios (S/N) for each element. Elemental species containing more than 40% of data < MDL were excluded from the PMF analysis. All elements were classified into three main groups, based on the signal to noise ratio (S/N): "strong" (S/N ≥ 2), "weak" (0.2 < S/N < 2), and "bad" (S/N ≤ 0.2). Although dawn-weighting was not applied for "strong" species, uncertainties were inflated by a factor of 3 for "weak" species. The third group treated as "bad" was excluded from the analysis. All species at NR-TOR were classified as "strong" with S/N > 2 (Table S3). As shown in Table S4, except for Se classified as "weak" with S/N of 1.2, all species at NR-VAN were classified as "strong" (S/N > 2).

Table S3. Summary of diagnostics for PMF with 4 factors for NR-TOR data.

	3-Factor Solution	4-Factor Solution	5-Factor Solution
Seed Value		Random	
# of Bootstraps Runs		100	
Minimum Correlation Value (R2)		0.8	
Displacement Active Species (S/N>2)		Al-XRF, Si-XRF, S-XRF, K-XRF, Ca-XRF, Ti-XRF, Fe-XRF, V, Cr, Mn, Ni, Cu, Zn, As, Se, Sr, Mo, Cd, Sn, Sb, Ba, La, Ce, Pb	
Bootstrap-Displacement Active Species		Si-XRF, K-XRF, Fe-XRF, Ba, La	
Factors with Bootstrap Mapping <100%	F2 (97%), F3 (98%)	none	F1 (73)
Displacement %dQ	-0.484	-0.014	-0.168
Displacement # of Swap	0	0	0
Bootstrap-displacement % of Case Accepted	98%	100%	93%
Bootstrap-displacement # of Swaps	1	0	6

Table S4. Summary of diagnostics for PMF with 4 factors for NR-VAN data.

	3-Factor Solution	4-Factor Solution	5-Factor Solution
Seed Value		Random	
# of Bootstraps Runs		100	
Minimum Correlation Value (R2)		0.8	
Displacement Active Species (S/N>2)		Al-XRF, Si-XRF, S-XRF, K-XRF, Ca-XRF, Ti-XRF, Fe-XRF, V, Cr, Mn, Ni, Cu, Zn, As, Sr, Mo, Cd, Sn, Sb, Ba, La, Ce, Pb	
Bootstrap-Displacement Active Species		Si-XRF, Fe-XRF, Cu, Ba, Ce	
Factors with Bootstrap Mapping <100%	F1 (98%), F2 (93%)	none	F3 (73), F5 (96%)
Displacement %dQ	−2.38	−0.006	−0.057
Displacement # of Swap	0	0	0
Bootstrap-displacement % of Case Accepted	97%	100%	83%
Bootstrap-displacement # of Swaps	0	0	4

US EPA PMF5.0 software accepts two types of uncertainty, including the observation-based and equation-based uncertainty. In this work, the equation-base uncertainty for data > MDL was applied as follows:

$$u_{ij} = (measured\ concentration\ \times \mathrm{ERU}) + \frac{\mathrm{MDL}}{3} \quad (6)$$

where ERU is the expanded relative uncertainty (Table S1).

Data below the MDLs were set to MDLs/2, with an uncertainty of 5/6 of the corresponding MDL. For the missing data, the median of all the concentrations measured for each element were used as its concentration, and its accompanying error were set at four times the median value so that it had minimal effect on the PMF results. Finally, an extra modeling uncertainty of 10% was implemented to both datasets to further reduce the noise within the data.

2.2. Justification of the PMF Solution

A large number of factors (2 to 10) were tested and 4 factors were found to yield the optimum results at for both data sets (Figure S1). No significant difference was estimated between the Q robust and true values (indicating that the model fit the outliers reasonably), and also between the Q true and theoretical values at the optimal solution. By conducting 100 bootstrap iterations, the percentage of factors assigned in each base case factor could be obtained. All runs provided very similar results indicated by the very low difference between the scaled residuals of the different runs. Furthermore, the standard deviation for the Q values over 100 runs was the smallest for the 4-factor solution, which also indicated the stability of this solution (Table S5).

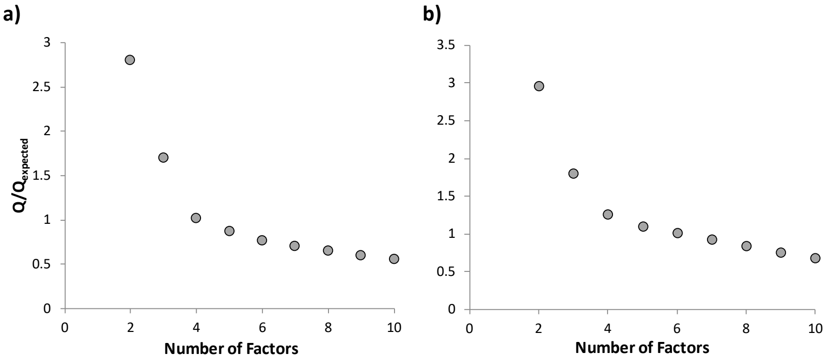

Figure S1. Inflection curves of the $Q/Q_{expected}$ value used to obtain the optimum number of PMF factors for **a**) NR-TOR and **b**) NR-VAN. An inflection at the 4-factor solution indicates that it is the most probable solution.

Table S5. Standard deviations (S.D.) of the Q-values (n=100) for NR-TOR and NR VAN.

Number of factors	NR-TOR	NR-VAN
2	396	211
3	140	158
**4**	**0.009**	**0.008**
5	21.5	26.6
6	33.5	8.8
7	4.31	16.4
8	1.89	14.1
9	5.48	17.3
10	6.58	21.9

The scaled residuals for all species in the 4-factor solution for both NR-TOR and NR-VAN datasets had normal distribution between −3 and +3, with the majority of them located between −2 and +2. Additionally, IM and IS experience drastic drop in their values at factor number 4 for both datasets (Figure S2). This indicated that the 4-factor solution for each dataset, NR-TOR and NR-VAN, was the optimal solution. The optimal number of factors for both sites were also assessed by the examination of the G-space plots, where they showed a reasonably good edges which indicates that the factors were independent from each other. In addition, the Fpeak analyses were conducted by changing the user-specified rotational parameter (Φ) between −0.4 and +0.4 and no change in the rotation was found to improve the results. Therefore, the value of Φ was set to 0.

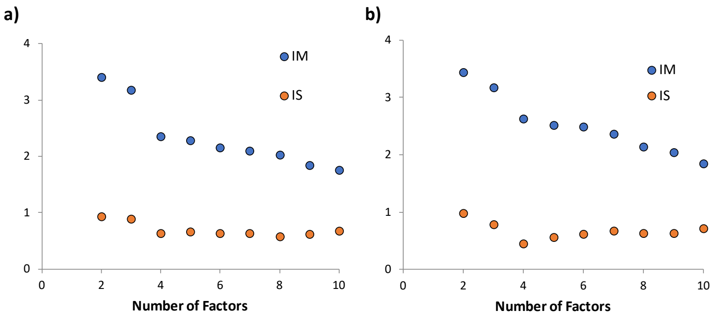

Figure S2. Determination of potential optimal number of factors by using the maximum individual mean and standard deviation for **a**) NR-TOR and **b**) NR-VAN.

In addition to the basic diagnostics, Bootstrap (BS) analysis, displacement (DISP) error estimation and bootstrap-displacement (BD-DISP) analysis of EPA PMF 5.0 were also conducted to ensure the optimal solution for each datasets (NR-TOR and NR-VAN). Via these three methods, the uncertainty of PMF analyses due to random errors and rotational ambiguity can be captured [2]. With BS of 100 iteration with minimum correlation R^2-

value of 0.8, the 4-factor solution showed that all factors were 100% mapped to the base factors which indicates the stability of the solution. In addition, the DISP showed that there was no factor swap and no drop in Q value, which indicates the 4-factor solution was reliable without any rotational ambiguity. Finally, BD-DISP results showed 100% mapping of all factor with minimal drop in the Q value (−0.014 for NR-TOR and −0.006 for NR-VAN) and no factor swap in the best fit and bootstrap-displacement at both NR-TOR and NR-VAN. The error estimation results for NR-TOR and NR-VAN confirmed that the 4-factor solution was stable, well defined, and physically meaningful. The diagnostic criteria for evaluating the PMF solutions in this study are summarized in **Tables S3–S5**

(a) (b)

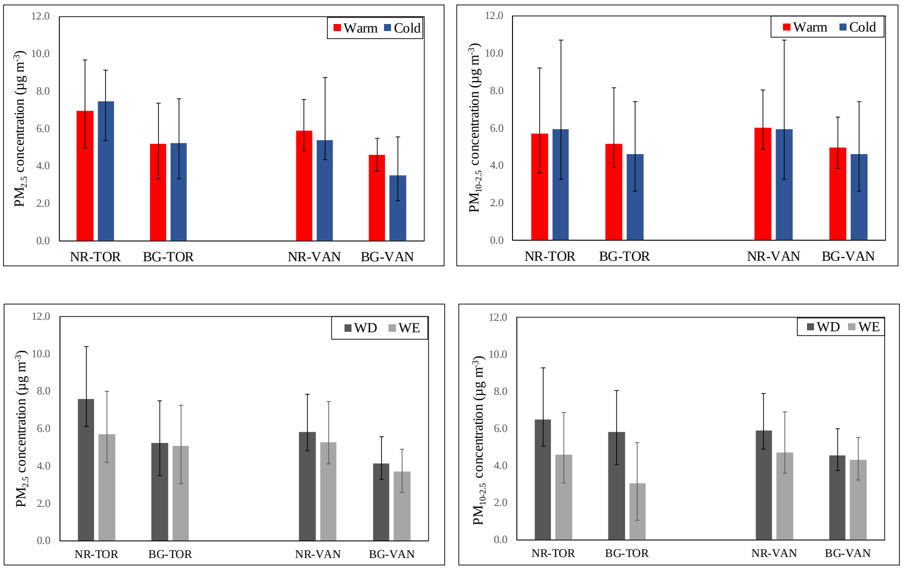

Figure S3. Median concentrations of $PM_{2.5}$ and $PM_{10-2.5}$ in (a) seasonal and (b) weekday/weekend collected samples over the Study Period listed in Table 1 (Main text). Whiskers represent IOR.

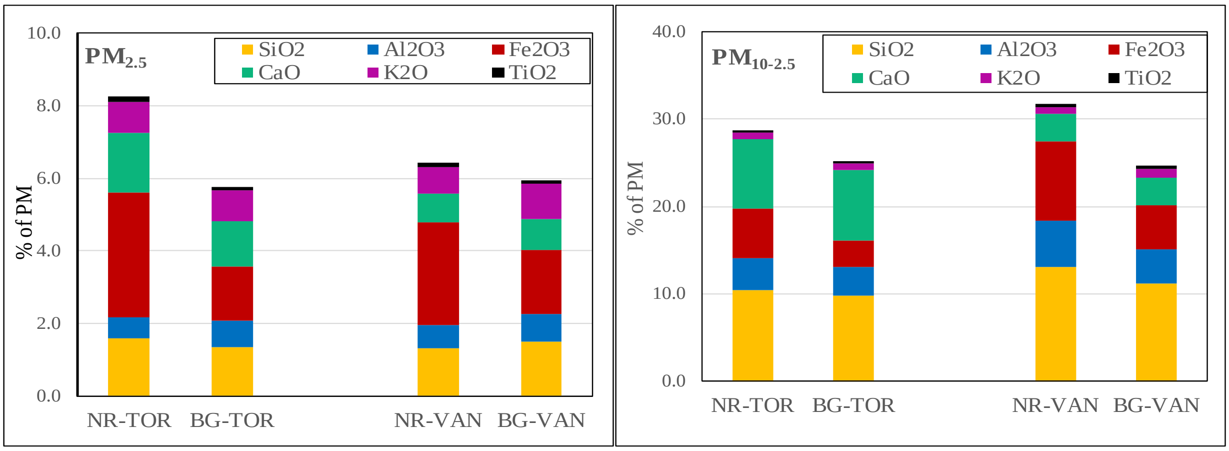

Figure S4. Contribution (in %) of crustal element oxides in $PM_{2.5}$ and $PM_{10-2.5}$ samples collected over the Study Period listed in Table 1 (Main text).

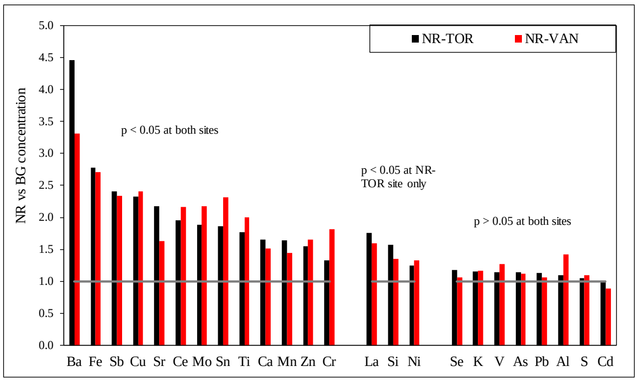

Figure S5. Median ratios (NR vs BG) of element concentrations. Kolmogorov-Smirnov test was used to compare concentrations of elements measured simultaneously at the near road and nearby background sites in Toronto and Vancouver.

(a) (b)

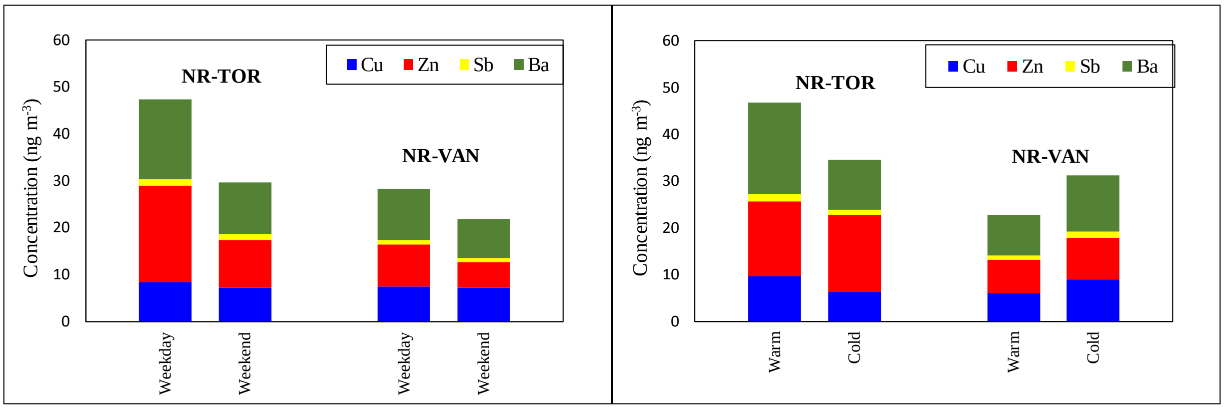

Figure S6. Median concentrations of brake-wear elements in (**a**) weekday/weekend and (**b**) seasonal $PM_{2.5}$ samples collected over the Study Period listed in Table 1 (Main text).

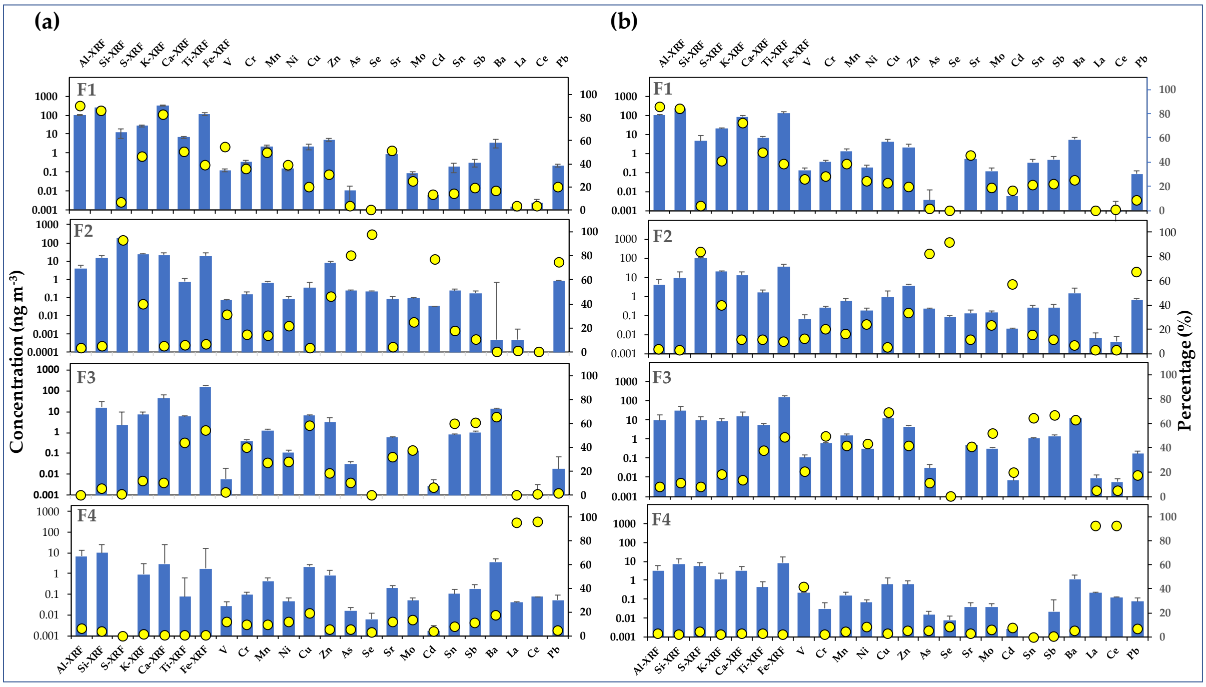

Figure S7. Source profiles of the PMF identified factors for a subset of fine and coarse PM samples analyzed for trace elements by ICP-MS: (**a**) NR-TOR and (**b**) NR-VAN. F1 = Mineral/Road Dust; F2 = Regional/Local Industry; F3 = Brake/Tire Wear; F4 = Unexplained.

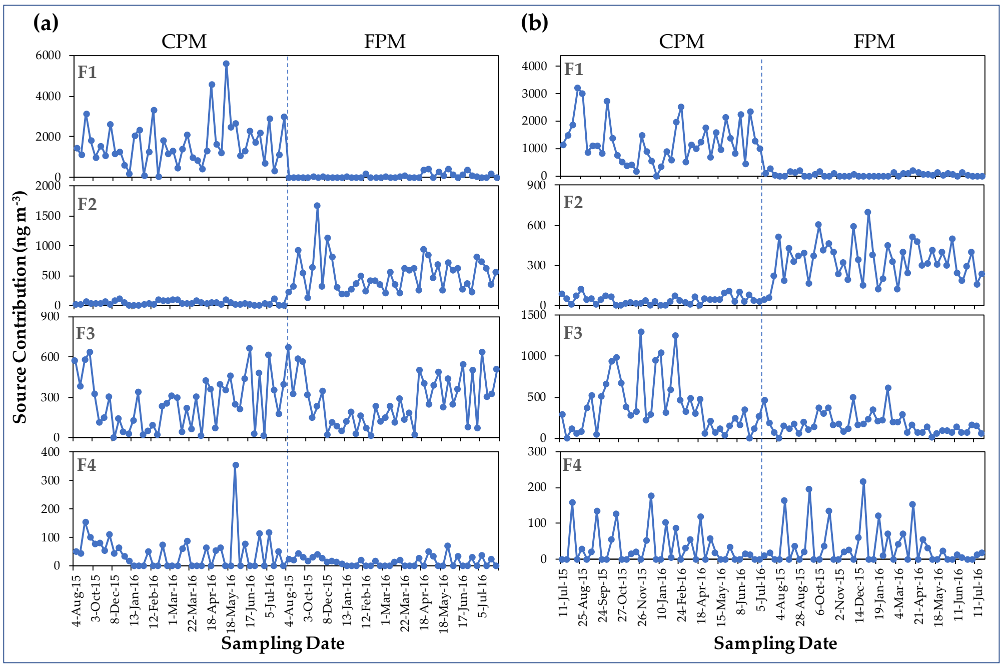

Figure S8. Time series of PMF resolved source factor contributions of metals in CPM and FPM for (**a**) NR-TOR and (**b**) NR-VAN.

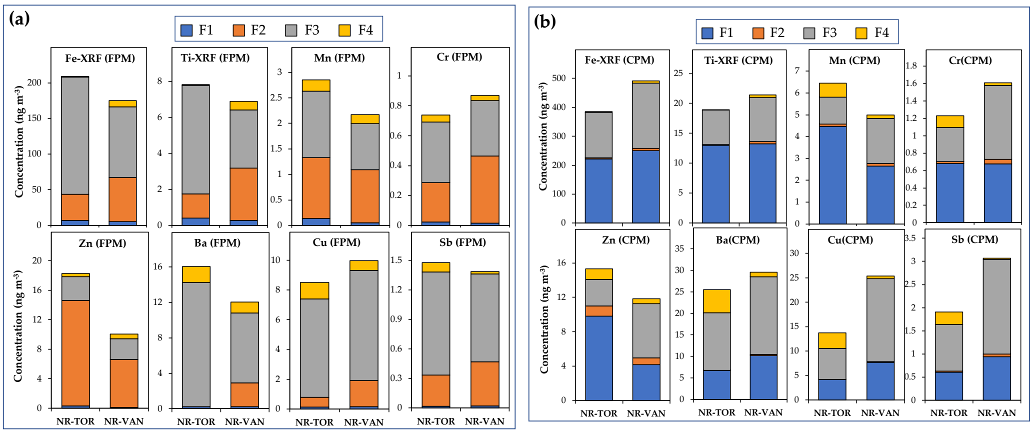

Figure S9. The absolute contribution of PMF-resolved sources to the concentration of individual redox-active metals in (a) $PM_{2.5}$ (FPM) and (b) $PM_{10-2.5}$ (CPM). F1 = Mineral/Road Dust; F2 = Regional/Local Industry; F3 = Brake/Tire Wear; F4 = Unexplained.

S3. References

1. Paatero, P.; Tapper, U. Positive matrix factorization: a non-negative factor model with optimal utilization of error estimates of data values. *Environmetrics* **1994**, 5, 111–126.
2. Brown, S.G.; Eberly, S.; Paatero, P.; Norris, G.A. Methods for estimating uncertainty in PMF solutions: Examples with ambient air and water quality data and guidance on reporting PMF results. *Sci. Total Environ.* **2015**, 518–519, 626–635.
3. Hopke, P.K. Review of receptor modeling methods for source apportionment. *J. Air Waste Manag. Assoc.* **2016**, 66, 237–259.
4. Juntto, S.; Paatero, P. Analysis of daily precipotation data by positive matrix factorization. *Environmetrics* **1994**, 5, 127–144.
5. Lee, E.; Chan, C.K.; Paatero, P. Application of positive matrix factorization in source apportionment of particulate pollutants in Hong Kong. *Atmos. Environ.* **1999**, 33, 3201–3212.
